# Feasibility and accuracy of 3D printed patient‐specific skull contoured brain biopsy guides

**DOI:** 10.1111/vsu.13641

**Published:** 2021-05-10

**Authors:** Richard Shinn, Clair Park, Kyrille DeBose, Fang‐Chi Hsu, Thomas Cecere, John Rossmeisl

**Affiliations:** ^1^ Department of Small Animal Clinical Sciences Virginia‐Maryland College of Veterinary Medicine, Virginia Polytechnic Institute and State University Blacksburg Virginia USA; ^2^ Animal Surgical Center of Michigan Flint Michigan USA; ^3^ Research Collaboration and Engagement University Libraries, Virginia Polytechnic Institute and State University Blacksburg Virginia USA; ^4^ Department of Biostatistics and Data Science, Division of Public Health Sciences Wake Forest School of Medicine Winston‐Salem North Carolina USA; ^5^ Department of Biomedical Sciences & Pathobiology Virginia‐Maryland College of Veterinary Medicine, Virginia Polytechnic Institute and State University Blacksburg Virginia USA

## Abstract

**Objective:**

Design 3D printed skull contoured brain biopsy guides (3D‐SCGs) from computed tomography (CT) or T1‐weighted magnetic resonance imaging (T1W MRI).

**Study Design:**

Feasibility study.

**Sample Population:**

Five beagle dog cadavers and two client‐owned dogs with brain tumors.

**Methods:**

Helical CT and T1W MRI were performed on cadavers. Planned target point was the head of the caudate nucleus. Three‐dimensional‐SCGs were created from CT and MRI using commercially available open‐source software. Using 3D‐SCGs, biopsy needles were placed into the caudate nucleus in cadavers, and CT was performed to assess needle placement accuracy, followed by histopathology. Three‐dimensional‐SCGs were then created and used to perform in vivo brain tumor biopsies.

**Results:**

No statistical difference was found between the planned target point and needle placement. Median needle placement error for all planned target points was 2.7 mm (range: 0.86–4.5 mm). No difference in accuracy was detected between MRI and CT‐designed 3D‐SCGs. Median needle placement error for the CT was 2.8 mm (range: 0.86–4.5 mm), and 2.2 mm (range: 1.7–2.7 mm) for MRI. Biopsy needles were successfully placed into the target in the two dogs with brain tumors and biopsy was successfully acquired in one dog.

**Conclusion:**

Three‐dimensional‐SCGs designed from CT or T1W MRI allowed needle placement within 4.5 mm of the intended target in all procedures, resulting in successful biopsy in one of two live dogs.

**Clinical Significance:**

This feasibility study justifies further evaluation of 3D‐SCGs as alternatives in facilities that do not have access to stereotactic brain biopsy.

## 1 INTRODUCTION

Intracranial neoplasia is a significant cause of morbidity and mortality in dogs, with a prevalence of 14.5 per 100 000 animals.^1^ With the increasing availability of magnetic resonance imaging (MRI), it is important to recognize the limitations for diagnosis as intracranial pathologies can share similar MRI signal characteristics and morphologies.^2^, ^3^, ^4^ For example, Cervera et al. found that up to 47% of cerebrovascular events were diagnosed as gliomas, with 12% of histologically confirmed gliomas classified as stroke.^4^ Ródenas et al. found that differentiation between neoplastic and non‐neoplastic lesions was possible in 89% of dogs with primary brain tumors, but was only correct in differentiating tumor type in 70% of primary brain tumors.^2^ As the treatment options and prognoses of each intracranial disease can significantly vary depending on the etiology, obtaining histopathologic diagnosis is a crucial step for both patients and clients.

In humans, brain biopsy is commonly performed as a stereotactic procedure.^5^ A frame‐based stereotactic brain biopsy (SBB), which is considered a gold standard for brain biopsies,^6^ utilizes a rigid external headframe to immobilize the patient, and a stereotactic coordination system to obtain the sample. Frame‐based SBB has been investigated in dogs and utilized effectively with precision and diagnostic yields comparable with human studies.^7^, ^8^, ^9^ However, several limitations, such as flexibility and patient discomfort, have been reported in people suggesting the need for different methodologies, including frameless techniques with robot‐assisted or image‐guided neuro‐navigation.^6^, ^10^ The use of stereotactic devices in veterinary medicine is further limited by the availability of the commercial devices and a wide range of patient sizes. Further investigation for alternative brain biopsy methods and stereotactic equipment can facilitate the diagnosis of brain lesions in dogs, and one potential alternative is a patient‐specific 3D printed biopsy guide. Patient‐specific 3D printed models and surgical guides have been used in veterinary medicine for various purposes with great success.^11^, ^12^, ^13^, ^14^, ^15^, ^16^, ^17^, ^18^ There are also two canine cadaveric studies that tested the feasibility of a 3D printed patient‐specific stereotactic system, but requires titanium bone anchors along with fiducial markers to be placed prior to MRI for planning of the 3D guide.^19^, ^20^

The goal of this study was to investigate the feasibility, describe the features, and test the accuracy of 3D printed patient‐specific skull contoured brain biopsy guides (3D‐SCG) in canine cadavers, and in dogs with spontaneous brain disease. We hypothesized that 3D‐SCGs could be designed based on MRI and computed tomography (CT), would be accurate for biopsy needle placement, and that CT‐designed 3D‐SCGs would be more accurate than MRI‐designed 3D‐SCGs, as the bone detail provided by CT would facilitate a better fit of the 3D‐SCG to the skull.

## 2 METHODS

### 2.1 Animals

All procedures performed in this study were approved by the Virginia Tech institutional animal care and use committees under separate protocols (19‐103‐VT & 20‐057‐VT) that approved the use of canine cadavers and client‐owned dogs. Initially, five beagle dog cadavers were used for evaluating the feasibility of 3D‐SCGs. The beagles were from the Virginia Maryland College of Veterinary Medicine teaching population and were euthanized for reasons not related to the study. Two client‐owned dogs were also included in the study. Owners of dogs provided written, informed consent to participate in the study.

### 2.2 Imaging studies

Helical CT (Toshiba Aquilion 16‐slice CT scanner, Japan) was performed on each of the five cadavers and one client‐owned dog prior to SBB. Following CT, two of the cadavers were randomly selected for MRI, and one client‐owned dog only had an MRI prior to SBB. T1 and 3D T1 MRI were performed on the two cadavers. Parameters for helical CT were as follows: field of view (FOV) was set at 512 × 512, slice thickness was set at 0.5 mm in transverse plane, cadavers were sternal, and a bone reconstruction algorithm was used. Magnetic resonance imaging was performed in‐hospital (1.5 T Philips Intera, Cleveland, Ohio) with an eight‐channel head coil. Parameters for T1 weighted (T1W) MRI were as follows: TE 11 ms, TR 300 ms, number of signals averaged 2, echo train length 1, flip angle 83°, transverse slice thickness 4 mm, interslice gap 4.4 mm, acquisition matrix 184 × 182, FOV 192 mm^2^. For 3D T1W MRI, parameters were as follows: TE 4.53 ms, TR 25 ms, number of signals averaged 2, echo train length 1, flip angle 30°, slice thickness 1.6 mm, interslice gap 0.8 mm, acquisition matrix 200 × 201, FOV 224 mm^2^. Images were transferred using Digital Imaging and Communications in Medicine (DICOM) format to a secondary workstation using open‐source medical image viewing software (Horos‐64bit version 3.3.6) for biopsy target planning, and for creating STL files. In the cadavers, the planned target point was the head of the caudate nucleus. To aid in visualization in the 3D modeling software, an approximately 2 mm^2^ circular region of interest (ROI) was created at the level of the head of the caudate nucleus for 3D‐SCG planning. For client‐owned dogs, an ideal trajectory was created for brain tumor biopsy avoiding larger vessels and as much gray matter as possible, and the center of the tumor was used as the planned target point.

### 2.3 3D printing of guides

**FIGURE 1:**
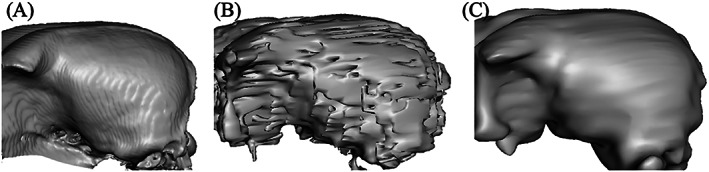
3D surface rendering of the skull based on the computed tomography (CT) (A), T1 magnetic resonance imaging (MRI) (B), and T1 MRI with Taubin smoothing (C)

All biopsy planning in cadavers was on the right side of the brain for CT 3D‐SCGs, and for MRI planning was on the left side of the brain. For CT, in the medical image viewing software, an STL file of the skull and ROI was created using the 3D Surface Rendering tool (Figure 1A). Settings were as follows: high resolution, 0.1 for decimate resolution, and 100 for smooth iterations. The STL file was then uploaded to the 3D modeling software Autodesk® Meshmixer (V3.5.474) for 3D‐SCG design. For MRI, the grow region tool was used for semiautomated selection of the skull. Lower threshold was set at 1, and upper threshold was set at approximately 180 for each cadaver. Once the segmentation parameters were adequate so that the skull was captured, the pixel value was set to 1000. An STL file was created using 3D Surface Rendering (Figure 1B). Settings were as follows: high resolution, 0.1 for decimate resolution, and 100 for smooth iterations. The STL file was then exported to 3D Slicer (V4.10.2) for Taubin smoothing, and a separate STL file was created (Figure 1C). This was performed to smooth out the edges on the MR images as shown comparing Figure 1B with Figure 1C. The STL files were then uploaded to the 3D modeling software for 3D‐SCG design.

**FIGURE 2:**
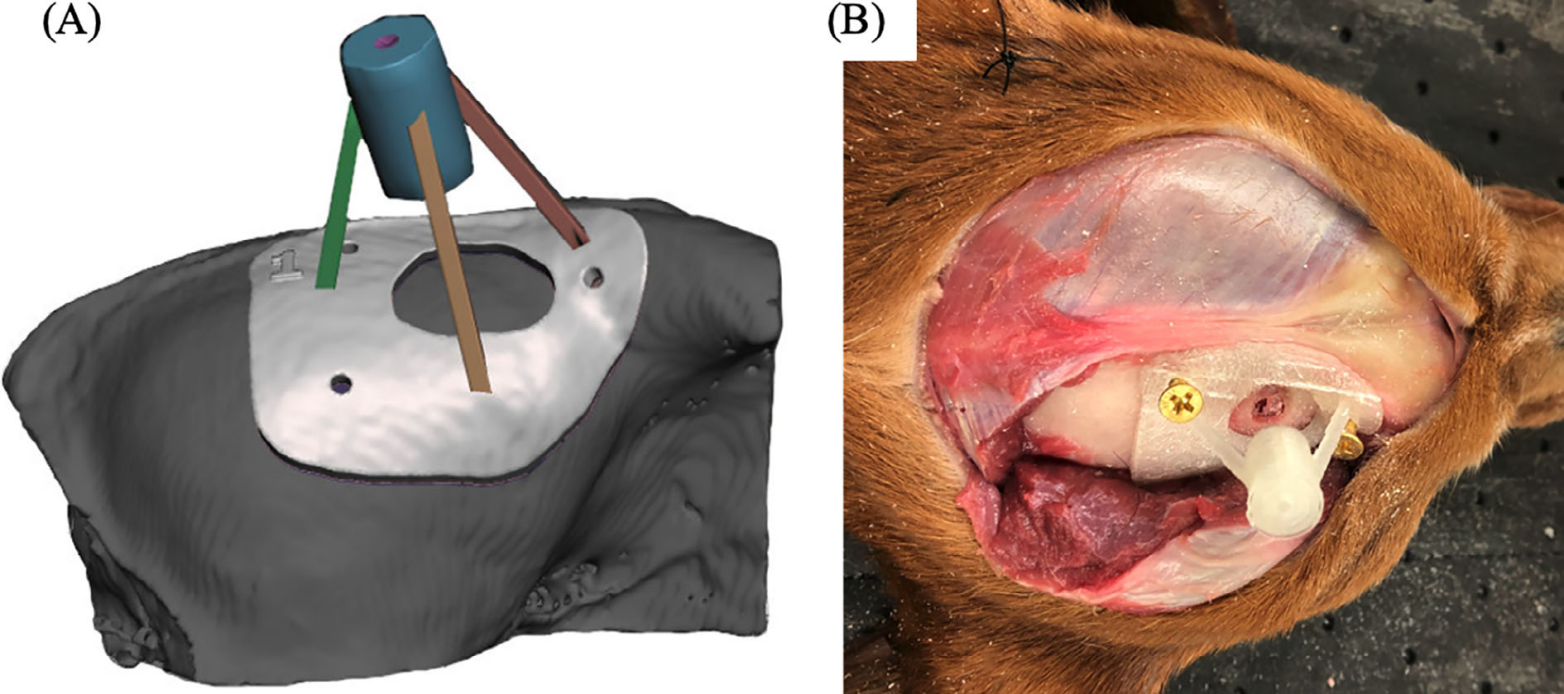
Example of a skull contoured 3D printed brain biopsy guide. (A) 3D rendering of the biopsy guide placed on the surface of the skull. The skull rendering was based on computed tomography. (B) 3D print of the biopsy guide anchored to the surface of the skull of a cadaver

In the 3D modeling software, a cylinder was created from the surface of the skull to the ROIs for each dog. The purpose of this cylinder was to function as a channel to house the biopsy needle and also serve to stabilize the needle. Cylinder length was then documented in mm from where it intersected with the ROI, to where it breached the surface of the skull. The cylinder was then extended by 30 mm from the surface of the skull. Using the 3D modeling software select tool, a footprint was created on the skull centered around the cylinder. The footprint was made large enough to have contact with the skull over multiple curvatures so that it would seat correctly and have room for screw placement, but not so large that it crossed midline caudal to the bregma or went so far rostral, ventral or caudal that it required muscle dissection other than elevation and reflection of the temporalis muscle laterally off the surface of the calvarium. Once selected, the footprint was extruded to be 3 mm in thickness. Using the Boolean difference tool, the channel in the cylinder was then hollowed out to the appropriate diameter to allow for shrinkage when printing. The plane cut tool was then used so the portion of the printed biopsy guide was 15 mm, leaving 15 mm exposed from the surface of the skull to allow for visualization of the brain when acquiring a biopsy. Supports were then created to connect the footprint of the skull to the cylinder (Figure 2A) and the 3D‐SCG was exported as an STL file. Initial 3D designs took approximately 6 h however after getting accustomed to the 3D modeling software, 3D designs took approximately 2.5 h.

The STL file was then exported to Cura Lulzbot Edition 21.04 3D for printing. The printer used was a Lulzbot Taz 6 with a 0.5 mm nozzle. PLA filament was used with a 2.85 mm diameter. The printer settings are as follows: layer height: 0.25, shell thickness: 1.0, bottom/top thickness: 1.0, fill density: 20%, print speed: 50 mm/s, printing temperature: 225°C, bed temperature: 60°C, supports: everywhere, overhead angle for support: 45°, fill amount: 15%, platform adhesion: none, flow: 100%. The average print time was approximately 4 h for each 3D‐SCG.

In client‐owned dogs, the CT‐based 3D‐SCG was designed in a similar fashion to cadavers except that two biopsy guides were created to give different possible trajectories into the tumor to account for vasculature that might not be visualized on CT. The MRI based 3D‐SCG was largely similar to the cadavers except that the upper threshold for the grow region tool was set at 280. Similar to the CT, MRI‐based 3D‐SCGs had three trajectories (Figure 4A). Client‐owned dogs also had 3D‐SCGs designed for convection enhanced delivery (CED) following tumor biopsy to deliver molecularly targeted therapeutics. The CED guide was created in a similar fashion to the CT‐based 3D‐SCG as described above but modified to fit the CED catheters (Figure 4B). Although CED delivery is not SBB, the CED procedure as well as design and placement of the 3D‐SCG are similar to SBB. The CED catheters were also visualized on MRI to acquire measurements for placement accuracy.

### 2.4 Surgical procedures

In cadavers, once the 3D‐SCG was printed, a standard (lateral) rostral tentorial craniectomy was performed.^21^ A midline linear incision was made through the skin from the caudal aspect of the frontal fossa to just rostral to the occipital protuberance (Figure 2B). The subcutaneous tissue was undermined and reflected ventrally. The temporalis muscle was incised in a curvilinear fashion from the zygomatic process of the frontal bone caudally until the 3D‐SCG was able to be placed on the skull (Figure 2B). A freer elevator was used to undermine the temporalis muscle and reflected ventrally to expose the frontal and parietal bones. The location of the craniectomy was determined by placing the 3D‐SCG on the skull temporarily, and tracing the desired craniectomy window size on the skull with an indelible surgical marker. Once the desired location of craniectomy window was determined, the 3D‐SCG was removed and a Sugairotome drill with a 4 mm oval burr was used to create the craniectomy defect. The 3D‐SCG was then placed on the skull and secured with 2 mm brass screws (Figure 2B). Brass screws were chosen as they are inexpensive, and cause minimal artifact on MRI or CT.

After the 3D‐SCG was secured to the skull, CT was performed on the skull of all five cadavers. A 20 gauge 3.5‐inch spinal needle (BD™ Quincke, Franklin Lakes, NJ) was then measured and placed through the 3D‐SCG into the brain. Computed tomography was then repeated so the needle location and trajectory could be measured. Afterwards, an equal parts mixture of permanent tissue marking dye (Davidson Marking System®, Bloomington, MN), iohexol (Omnipaque™ 300 mg/ml), and gadoteridol (ProHance® 0.5 mmol/ml) was injected through the needle. A total of 0.3 ml was injected into each needle. Computed tomography was then repeated to view the location of the iohexol. Magnetic resonance imaging was also performed following CT to identify the location of the gadoteridol.

**FIGURE 3:**
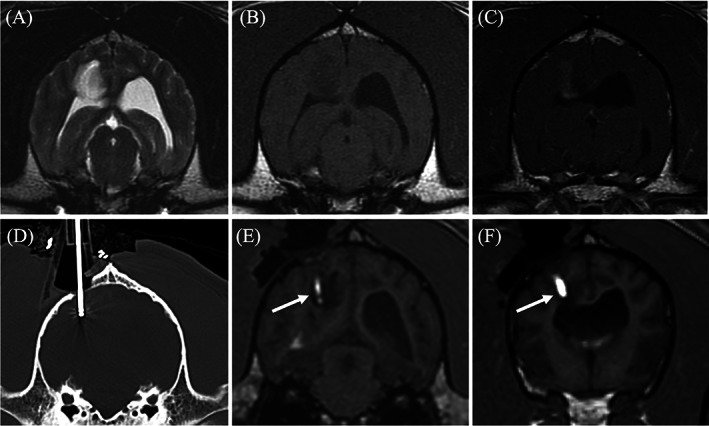
Transverse diagnostic imaging of a live dog. (A) T2 weighted transverse image. A hyperintense mass is present within the right parietal lobe. (B) The mass appears hypointense on T1 weighted (T1W) images and has moderate heterogeneous contrast enhancement (C). (D) Transverse computed tomography (CT) set on a bone window showing the placement of the biopsy needle within the mass. (E) Transverse 3D T1W image during convection enhanced delivery (CED) of molecularly targeted therapeutics into the mass. The arrow is displaying the therapeutics which is infused with gadolinium making the needle placement and trajectory visible on magnetic resonance imaging. (F) A more rostral transverse 3D T1W image compared to (E) where a separate needle was placed. The CED guide allowed for multiple needles to be placed within the mass for a more even distribution of the therapeutics

**FIGURE 4:**
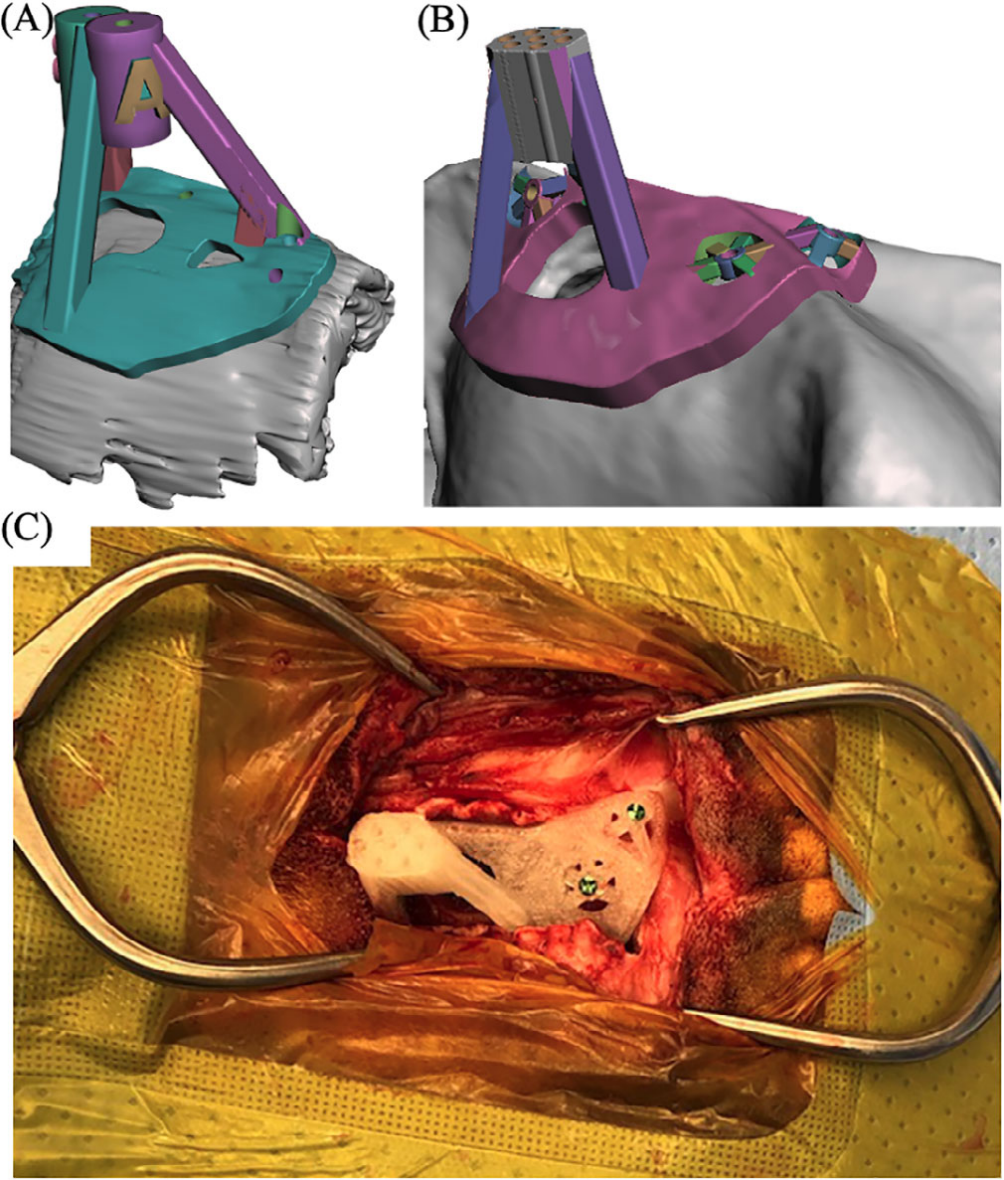
Example of a skull contoured 3D printed guide applied to a live dog. (A) 3D rendering of the biopsy guide placed on the surface of the skull. The skull was based on transverse T1 weighted images. (B) 3D rendering of the convection enhanced delivery (CED) guide placed on the surface of the skull. The skull rendering was based on computed tomography. (C) 3D print of the CED guide anchored to the surface of the skull

Client‐owned dogs were anesthetized with a benzodiazepine and fentanyl for premedication and induced with propofol intravenously. Following induction and intubation all dogs were placed in sternal recumbency. Propofol was used for anesthetic maintenance in combination with fentanyl or remifentanil. The head of each dog was clipped then prepped aseptically for surgery. A standard (lateral) rostral tentorial craniotomy was performed as described above. Cefazolin was given 30 min prior to starting the incision then continued every 90 min thereafter (22 mg/kg IV). After enough of the skull was exposed, the 3D‐SCG was fixed to the skull using 2.0 mm self‐tapping titanium screws. All 3D‐SCGs were gas sterilized with ethylene oxide over 17 h prior to SBB. A Laitinen biopsy needle (16‐gauge side‐cutting aspiration biopsy needles with a 10‐mm cutting channel, Integra Radionics, Burlington, Massachusetts) was then inserted into the mass to obtain a biopsy. Then CT was performed while the biopsy needle was in place (Figure 3B) to ensure proper positioning of the needle. Following the biopsy procedure, the 3D‐SCG was removed and the incision was closed until the CED guide could be created from the CT scan. The dog was taken back to surgery and the CED guide was fixed to the dog using 2.0 mm self‐tapping titanium screws (Figure 4C). While the CED guide was fixed to the dog, the CED needles were placed into the tumor to deliver molecularly targeted therapeutics. The therapeutics contain gadoteridol contrast which allowed visualization of the CED catheters on MRI (Figure 3E). Following delivery of the therapeutics and MRI, the CED guide was removed, a titanium mesh cranioplasty was performed, and the incision was closed.

### 2.5 Cadaver and biopsy processing

Following imaging and surgery on cadavers, all brains were collected and fixed in 10% neutral buffered formalin for a minimum of 72 h at room temperature. After formalin fixation, transverse sections were made through the brain at the level of the caudate nucleus to visualize the dye. Once the dye was visualized, the transverse sections were paraffin‐embedded, sectioned in 2–5 mm slices, mounted on glass slides, and routinely stained with hematoxylin and eosin (H&E). All gross and histologic analyses were performed by a single board‐certified veterinary anatomic pathologist.

In live dogs, biopsy specimens were fixed in 10% neutral buffered formalin for a minimum of 24 h then paraffin‐embedded, and 5 μm sections were stained with H&E and murine monoclonal antibody against glial fibrillary acid protein. At least two board‐certified veterinary pathologists reviewed the biopsies.

### 2.6 Data analysis

To assess the accuracy, the planned target point was compared to the tip of where the needle was placed.^22^ To summarize, the planned target point image was fused with the needle placement image. Once aligned, images were viewed in sagittal and a perpendicular line was drawn from the ROI (planned target point or needle tip) to the caudal aspect of the occipital protuberance to obtain the *Z* (planned target point) and *Z*′ (needle tip) measurement. Images were then viewed in transverse and a right angle was drawn from the ROI vertically to the inner calvarium and horizontally to obtain the *Y* and *Y*′, and *X* and *X*′ measurements respectively. Needle placement error was then calculated as follows: √[(*X* − *X*′)^2^ + (*Y* − *Y*′)^2^ + (*Z* − *Z*′)^2^].

To compare the planned target to the needle placement, a paired *t*‐test was used since the difference was normally distributed. Spearman's rank correlation was used to relate target point to needle placement error. Median needle placement error and range were then calculated for all dogs, dogs where a 3D‐SCG was designed from CT, and dogs where a 3D‐SCG was designed from MRI. A paired *t*‐test was used to compare needle placement error between CT and MRI‐designed 3D‐SCGs. Analysis and graphic generation of data was performed using JMP® (Pro 14.0.0) statistical software. *p*‐Values of .05 or less were considered statistically significant.

## 3 RESULTS

**FIGURE 5:**
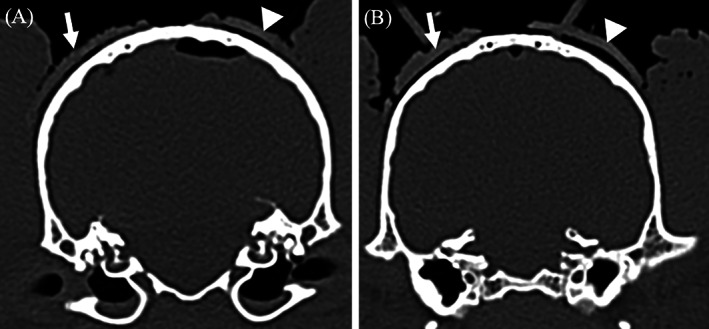
Comparison of computed tomography (CT) and T1 weighted (T1W) designed 3D printed skull contoured brain biopsy guides (3D‐SCGs). (A) The arrow is outlining the outer edge of the CT‐designed 3D‐SCG where the arrow head is outlining the outer edge of the T1W designed 3D‐SCG. The distance from the skull to the inner edge of the CT and TW1‐designed 3D‐SCG is 0.73 and 1.14 mm respectively. (B) Similar to (A), the arrow is outlining the outer edge of the CT‐designed 3D‐SCG where the arrow head is outlining the outer edge of the T1W‐designed 3D‐SCG. The distance from the skull to the inner edge of the CT and T1W‐designed 3D‐SCG is 0.8 and 1.41 mm respectively

In total, 13 different needles were placed using 3D‐SCGs in five cadavers and in two live dogs. The live dogs were a 7‐year‐old male neutered (MN) English bulldog, with a high‐grade oligodendroglioma located within the parietal lobe measuring 1.16 cm^3^, and an 8‐year‐old MN Boxer with a high‐grade astrocytoma primarily located within the piriform lobe measuring 8.3 cm^3^. On MRI, both tumors were hyperintense on T2‐weighted images, hypointense on T1‐weighted images, and had minimal contrast enhancement. A 3D‐SCG was able to be designed for each cadaver and live dog and was able to be placed on the skull of each dog. The CT planned 3D‐SCGs seated well with the skull however the MRI planned 3D‐SCGs were raised from the skull (Figure 5).

**FIGURE 6:**
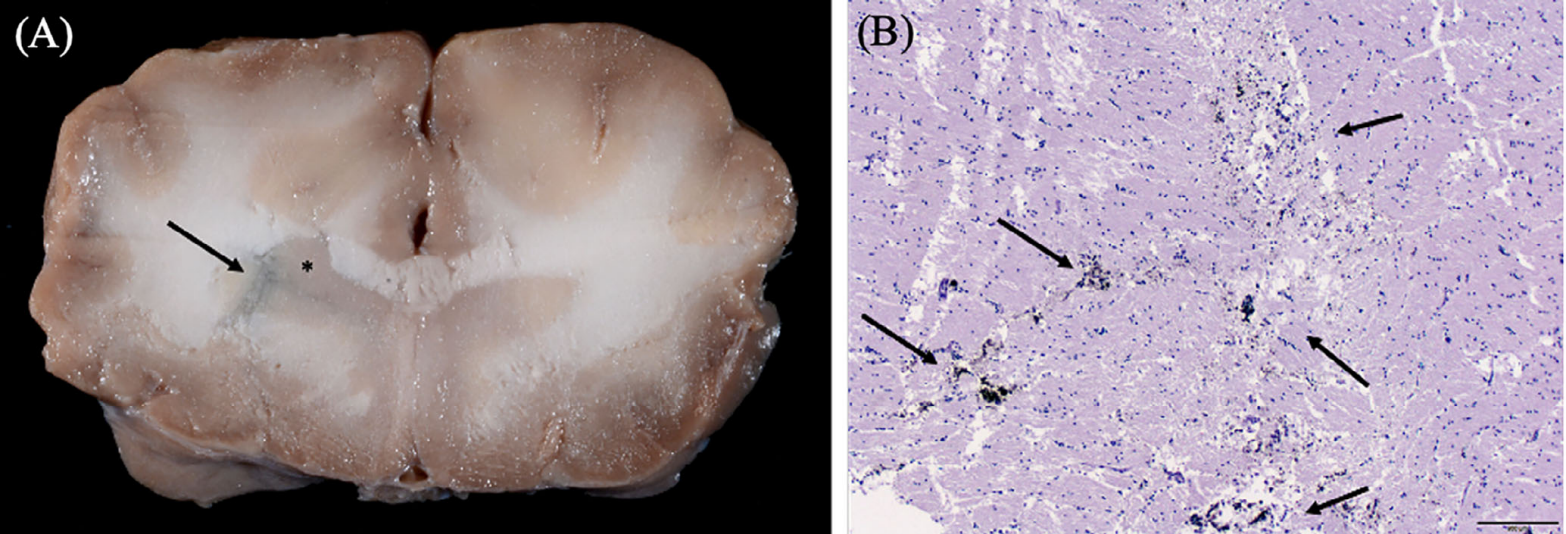
Gross and histopathologic images of cadaver samples. (A) Gross transverse image of the brain at the level of the head of the caudate nucleus as indicated by the asterisk. The tissue marking dye is in and around the caudate nucleus as depicted by the arrow. (B) Histopathologic H&E section at 40× magnification at the level of the caudate nucleus. The dye is visualized within the section as indicated by the arrows

On gross specimens, tissue marking dye was visualized in and around the caudate nucleus in four of the five cadavers (Figure 6A). The cadaver where the tissue marking dye was not visualized had too much autolysis of the brain parenchyma to decipher where the caudate nucleus was located. Only one of the cadavers had diagnostic quality images obtained from histopathologic samples due to autolysis and freeze/thaw artifact (Figure 6B).

**FIGURE 7:**
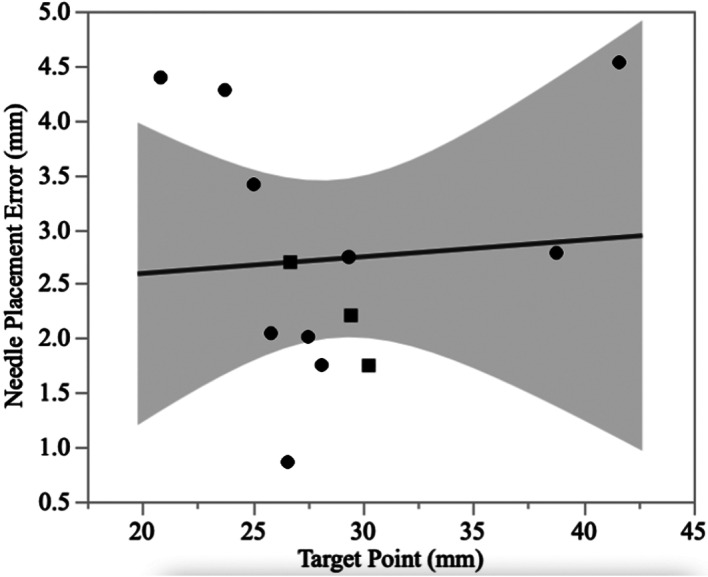
Comparison between the measured biopsy needle length and the absolute target point difference for both magnetic resonance imaging (MRI, squares) and computed tomography (CT, circles) designed guides. The solid line represents the line best fit, and the shaded area represents the 95% CI

**FIGURE 8:**
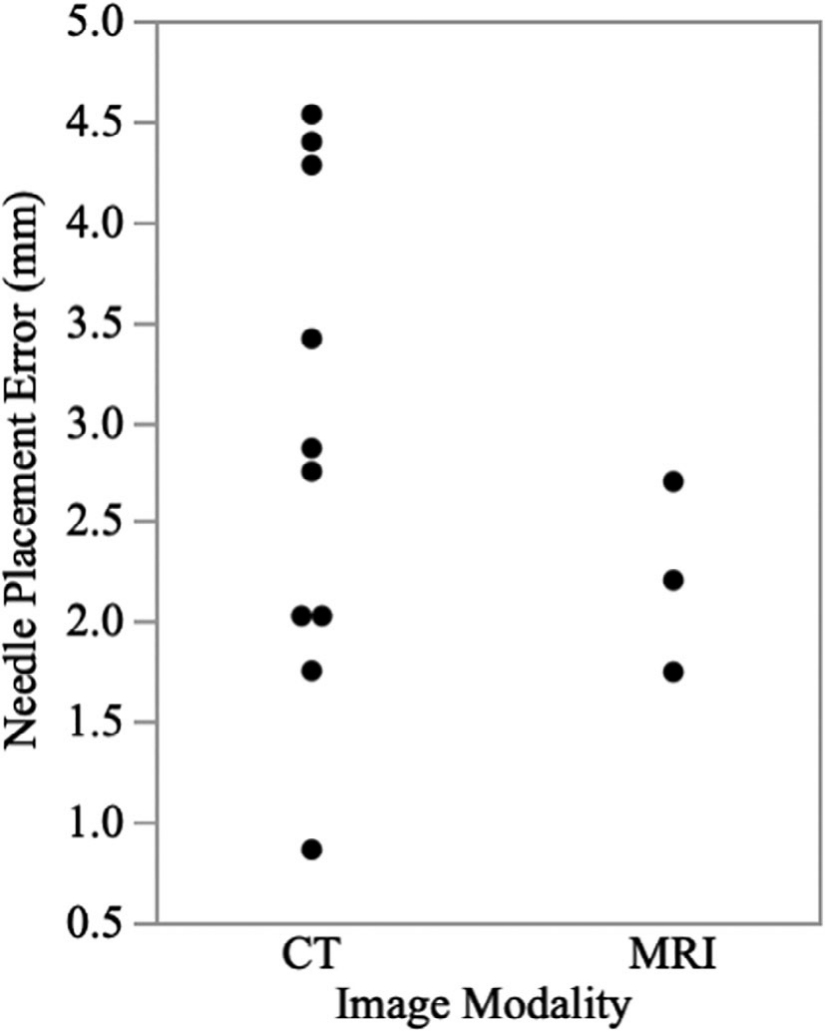
Scatter plot showing the absolute target point difference between magnetic resonance imaging (MRI) and computed tomography (CT)

The median needle placement error was 2.7 mm (range: 0.86–4.5 mm). Overall, there was no significant difference between the planned target point and the needle placement (*p* = .17). When comparing the target point to needle placement error (Figure 7), no correlation was found (*r* = .005, *p* = .8). When comparing CT 3D‐SCGs (*n* = 10) to MRI 3D‐SCGs (*n* = 3), there was not a significant difference in accuracy (*p* = .98, Figure 8). The median needle placement error for the CT was 2.8 mm (range: 0.86–4.5 mm), and the median needle placement error for the MRI was 2.2 mm (range: 1.7–2.7 mm).

In client‐owned dogs, no complications were encountered during the biopsy procedures. The biopsy obtained in one dog was a large volume and of diagnostic quality. In the other dog, although the placement of the Laitinen biopsy needle was accurate, due to lack of negative pressure from the Laitinen needle, biopsy was not acquired. This dog was subsequently successfully biopsied under the same anesthetic episode using a freehand biopsy technique.^23^ Delivery of therapeutics was deemed successful, and both dogs recovered without incident from the procedures.

## 4 DISCUSSION

In this study, the feasibility and accuracy of 3D‐SCGs were evaluated in canine cadavers and in two clinical cases. A 3D‐SCG was able to be designed for each of the dogs despite variations in skull size and shape. No significant difference was found between the planned target point and needle placement. Accuracy of MRI and CT‐designed 3D‐SCGs appear to be similar.

There is no shortage of SBB techniques in the dog. The first SBB technique was described in the dog in 1982.^24^ Since then, over a dozen different SBB techniques have been evaluated.^7^, ^19^, ^22^, ^25^, ^26^, ^27^, ^28^, ^29^, ^30^, ^31^, ^32^, ^33^, ^34^, ^35^ When reported, the mean or median needle placement error range is 0.83–4.3 mm.^7^, ^22^, ^29^, ^30^, ^33^, ^34^, ^35^ For non‐cadaver studies, biopsy has been acquired in 80%–95% of cases where SBB was performed.^7^, ^27^, ^29^, ^31^ Our median needle placement error was similar to previous reports. We were able to obtain a biopsy in only one of two live dogs. Although this could be related to the 3D‐SCG, it is more likely related to planning error. The tumor where a biopsy was not acquired was a superficial tumor and the planned needle depth was only 9 mm. In order to create a negative pressure seal, the Laitinen biopsy needle needs to be 10 mm into the tissue. This stresses the importance of individualized planning and planning verification when executing SBB. On CT, the needle was placed at the desired location but because a negative pressure seal was not created, we were not able to obtain a biopsy until the Laitinen biopsy needle was placed deeper into the tumor. Koblik et al. had a similar finding where all but one dog was diagnosed on SBB, which was a dog with a superficial brain tumor.^29^

To the author's knowledge, this is the first study to design a SBB guide based solely on MRI without fiducial markers or a neuronavigation system. We elected to include this in our study as there are times when CT might not be available. Although CT has greater measurement accuracy of tissue compared to MRI,^36^ in people MRI has been shown to fall within acceptable ranges for SBB.^37^ We found the accuracy of MRI‐designed 3D‐SCG to be similar to CT; however, this technique should be used with caution for a number of reasons. First, when the MRI‐designed 3D‐SCG was applied, it was obvious that it did not seat perfectly to the contour of the skull (Figure 5). The MRI‐designed 3D‐SCGs were applied to two beagles, and one English bulldog. The shape of the beagle skull is mesaticephalic, and the skull of the English bulldog is quite thick making visualization of the skull on MRI straightforward. For breeds where the skull is not as easily visualized, or if the skull conformation is not normal, MRI‐designed 3D‐SCGs might be even less form fitting. Secondly, MRI‐designed 3D‐SCGs were only performed for three needle placements and a type II statistical error is possible. Third, the Taubin algorithm was chosen as it does not change the surface dimensions but it has not been verified for medical applications.^38^ Despite this, the Taubin algorithm was necessary in order for the MRI‐designed 3D‐SCG to seat on the skull with any uniformity. Ideally, a larger investigation should be made into the use of the Taubin algorithm for medical applications. Finally, T1W images were used in this study as it is not possible to visualize bone well enough on T2 weighted images. Newer techniques are available, such as 3D‐T1W images and Black Bone, which would improve visualization of the skull. Ideally, thinner slices or bone‐specific techniques should be used for MRI‐designed 3D‐SCG when CT is not available.

In veterinary medicine, there is no consensus on what constitutes a clinically acceptable application accuracy for SBB. The definition of acceptability may also vary depending on the type and location of the lesion being sampled.^29^, ^30^, ^34^ Previously published accuracies have had considerable variability (overall application accuracies ranging from 0.83–3.6 mm), and not all investigations of SBB in the dogs have quantified application accuracy.^22^, ^29^, ^30^, ^35^ When evaluated for the clinical diagnosis of naturally occurring neoplastic brain lesions in dogs that exceed 5 mm in diameter, application accuracies of 1.5–3.5 mm have resulted in diagnostic yields in >91%, which is similar to humans, suggesting this range is acceptable for clinical use in dogs.^7^, ^29^ A target error of less than 3 mm has also been stated as the minimum requirement for SBB,^33^ with reported upper error limits of 3.4, 3.6, 3.9, and 4.6 mm.^7^, ^29^, ^30^, ^34^ Therefore, the accuracy of 3D‐SCGs reported here falls within the ranges of other SBB techniques. A recent study investigating MRI‐based SBB techniques in dogs provides evidence that MRI techniques are capable of submillimeter application accuracies, but there are currently no studies providing direct head‐to‐head performance comparisons of SBB techniques in dogs with spontaneous brain lesions.^19^ It is also difficult to identify possible reasons for differences in application accuracy or precision between SBB techniques in dogs, as published data regarding sources contributing to clinically relevant error that are encountered throughout the SBB process, such as image registration, target point selection and vector calculation, headframe mechanical specifications, intraoperative brain deformation, and operator (human) factors are not available.^19^, ^22^, ^29^, ^30^, ^35^

There are several key benefits of the 3D‐SCG compared to previously evaluated SBB techniques. The 3D‐SCG does not require special equipment other than a desktop 3D printer and commercially available filament. It is also possible for a third party to design, print, and mail the 3D‐SCG relinquishing the need for any additional equipment at most veterinary facilities. This could help save on the initial cost compared to other SBB techniques. Also, surgery or positioning in a head frame is not required prior to design of the 3D‐SCG potentially limiting the number of anesthetic events. Real‐time imaging with CT can also be used with 3D‐SCGs to ensure proper placement of the needle into the desired location as artifact from the 3D‐SCG is absent other than the screws holding the 3D‐SCG in place.

There are also limitations to the 3D‐SCG. The most obvious drawback is that it takes time to design and print the 3D‐SCG and knowledge on how to use the various software platforms. Even after getting accustomed to the methods, approximately 2.5 h were needed to design a single 3D‐SCG. In addition, a single print takes approximately 4 h but does vary depending on the size of the 3D‐SCG. The 3D‐SCG must also sit directly on the skull making a small myotomy impossible. We also elected to use food grade PLA filament as it is FDA approved for pharmaceutical and medical applications, is biodegradable, and has minimal shrinkage compared to other materials.^39^ A disadvantage of PLA is that it will decrystallize between 95°C and 115°C depending on the l‐lactide content and the type of crystals formed during the crystallization meaning it cannot be autoclaved.^40^ Low‐temperature sterilization is therefore needed such as with ethylene oxide or hydrogen peroxide plasma sterilization. This could be a limiting factor at certain facilities where low‐temperature sterilization is not available. Another limitation is that the trajectory cannot be changed once the 3D‐SCG is designed. This limitation was highlighted in the one live patient where a biopsy was not acquired due to the cutting channel of the biopsy needle not being deep enough into the tumor. Finally, although the 3D‐SCG is designed to fit the skull specifically in one specific manner, it is still possible for the alignment to be off if not fitted properly. This could be especially important for tumors deep within the brain parenchyma.

There are also limitations of the study itself. First is the limited number of live dogs in the study and the number of biopsy samples. Although an attempt was made to confirm needle placement histopathologically in cadavers, the degree of autolysis and freeze/thaw artifact made it impossible to confirm the location of the tissue marking dye in the majority of samples. Fusion imaging was performed to evaluate needle placement compared to the planned target point, but target registration error was possible as the ROI coordinates were taken manually rather than with automated software. The images were fused to try and limit this error but without having a fiducial marker, there was still the possibility of human error. Finally, some of the measurements were done on CT for planning, while the needle placement measurements were done on MRI. This could alter the results as MRI measurements can differ slightly from that of CT.^30^

In conclusion, 3D‐SCGs designed from CT or MRI allowed needle placement within 4.5 mm of the intended target in all procedures, resulting in successful biopsy in one of two live dogs. The proposed system is feasible and justifies further evaluation of 3D printed skull contoured brain biopsy guides as alternatives in facilities that do not have access to SBB. The significant reduction in size and weight of the system compared to previously described biopsy instruments, coupled with the acceptable accuracy in needle placement, make the current design promising. Further studies are needed to characterize the diagnostic accuracy, diagnostic yield, reproducibility, and reliability of the system.

## CONFLICT OF INTEREST

The authors declare no conflict of interest related to this report.

## AUTHOR CONTRIBUTIONS

Richard Shinn proposed the project, contributed to the conception of the study design and acquisition of the data, the design of 3D Printed Skull Contoured Guides, facilitated in surgery for cadavers and patients, and to all stages of manuscript authoring and revision. Clair Park contributed to the conception of the study and drafting of the initial manuscript. Kyrille DeBose contributed to the conception of the study, the design and printing of the 3D Skull Contoured Guides, and final manuscript authoring and revision. Fang‐Chi Hsu contributed to the analysis and interpretation of data for the work. Thomas Cecere read and interpreted gross and histopathologic samples acquired. John Rossmeisl contributed to the conception of the study design, facilitated in surgery for cadavers and patients, and to all stages of manuscript authoring and revision.
